# Esketamine multi-omic biomarker evaluation in major depressive disorder (EMBER-MDD): concept, objectives and methodologies of a non-clinical investigator-initiated study

**DOI:** 10.1007/s00406-026-02306-x

**Published:** 2026-06-30

**Authors:** C. Hohoff, T. Lange, L. Steinbach, S. M. Wissing, E. Van Assche, B. T. Baune

**Affiliations:** 1 Department of Psychiatry, University of Muenster, Muenster, Germany; 2 Institute of Medical Data Science, Otto-von-Guericke-University Magdeburg, Magdeburg, Germany; 3 Department of Psychiatry, Melbourne Medical School, University of Melbourne, Melbourne, Australia; 4 Department of Psychiatry, The Florey Institute of Neuroscience and Mental Health, Melbourne, Australia

**Keywords:** Treatment resistance, Depression, Esketamine, Molecular omics, Integrative multi-omics

## Abstract

Treatment resistance (TR) in major depressive disorder (MDD) affects a substantial minority of patients and is hard to recognize early, delaying intensified care. The Esketamine multi-omic biomarker evaluation in MDD (EMBER-MDD) is a non-interventional, investigator-initiated, in-vitro study within the EU Psych-STRATA programme, analyzing biospecimens collected in the randomized INTENSIFY study and the mirror OBS-TR cohort after participants complete treatment. EMBER-MDD aims to discover individual-omic and integrated multi-omic (hypothesis-free) biomarkers and signatures associated with TR risk, and molecular correlates of clinical response to esketamine nasal spray versus treatment as usual (TAU). Biomaterials will derive from approximately 420 adults with MDD (estimated *n* = 210 esketamine; *n* = 210 TAU) and include whole blood, RNA-stabilized whole blood, plasma and serum, sampled at baseline and, when feasible, during and after treatment (up to ~ 5,040 aliquots stored at − 80 °C). Genomics will use baseline DNA genotyping on Illumina Infinium GSA v3.0+MD arrays; epigenomics will profile genome-wide DNA methylation across time points using MethylationEPIC v2.0; transcriptomics will employ mRNA-seq (NovaSeq X/ X Plus); and proteomics/ metabolomics will be generated using high-throughput Olink and/ or Biocrates platforms. Each layer will undergo state-of-the-art preprocessing and analyses (e.g., GWAS/ PRS, EWAS, differential expression, WGCNA, pathway and network analyses), followed by integrative strategies including QTL mapping (meQTL/ eQTL/ pQTL/ mQTL) and intermediate-fusion machine learning with nested cross-validation, explainable AI (SHAP/ LIME) and treatment-effect modelling. All outputs are research-only and will not support individual efficacy, tolerability, or clinical decision-making. The study will deliver robust biosignatures and mechanistic hypotheses to guide future validation and inform stratified, molecularly guided intervention strategies in subsequent prospective trials. Trial registration number: 2023-506617-21–00 and 2025-178-f-S.

**Supplementary Information:**

The online version contains supplementary material available at https://doi.org/10.1007/s00406-026-02306-x.

## Background

Mental illnesses, such as major depressive disorder (MDD), Schizophrenia (SCZ), and bipolar disorder (BD), are common and severe. In about one third of cases, associated patients suffer from resistance to therapy, risking a chronic course of illness with manifest loss of quality of life [1]. Early signs of treatment resistance (TR) are hard to reliably define, leading to a delayed adequate treatment with intensive pharmacological therapy for those at risk for a chronic course of illness. The current multidisciplinary EU-funded project Psych-STRATA (A Stratified Treatment Algorithm in Psychiatry: A program on stratified pharmacogenomics in severe mental illness; [2]), addresses this problem of identification of individuals at risk for treatment resistance and finding a fitting treatment strategy. Psych-STRATA aims at (a) investigating the molecular basis of TR in severe psychiatric illnesses, (b) identifying patients at risk of TR at an early stage and (c) treating such patients with intensified pharmacological treatment strategies. Psych-STRATA zooms in on three major psychiatric disorders, SCZ, BD and MDD. It consists of seven work packages, which comprise the identification of multimodal biosignatures associated with TR, response and an early intervention in SCZ, BD and MDD, using an intensified treatment paradigm compared to treatment as usual (TAU). Overall, Psych-STRATA aims to develop a molecularly informed prediction tool to identify patients at risk for TR. One sub-project of Psych-STRATA deals with the treatment of patients as part of a clinical trial. This clinical trial, “INTENSIFY”, is an in-vivo study with randomized controlled setting and early intensified pharmacological treatment compared to TAU. For each patient, INTENSIFY treatment is intended to last six weeks, with follow-up up to six weeks later, but can be stopped earlier or last longer dependent on the patient’s requests. In MDD, patients with TR risk are treated with either another oral antidepressant plus additional esketamine nasal spray or with a conventional second-line antidepressant (TAU) as described in detail in the Psych-STRATA study protocol [2]. During INTENSIFY treatment, blood samples are collected to gain different biomaterials, at least at begin of treatment (baseline) and, if possible, during and after treatment.

The present Esketamine multi-omic biomarker evaluation in MDD (EMBER-MDD) study aims to contribute further to the above-mentioned Psych-STRATA goals with in-depth exploration of collected biomaterials of MDD patients at risk for TR (patients who failed to respond to a first-line therapy in the current disease episode, analogue to Psych-STRATA [2]). The EMBER-MDD study brings together multiple molecular layers of information, to identify the molecular signature of TR risk particularly in MDD patients. It is an in-vitro study that focuses on laboratory and bioinformatic analyses based on biomaterials obtained from MDD patients, after these patients completed their treatments (switched to either another oral antidepressant plus esketamine (ESK) or to a second line antidepressant (TAU)). Biomaterials originate from INTENSIFY and its exact mirror study “OBS-TR” (localized in Münster, Dept. of Psychiatry, University of Münster, Germany), after the MDD patients left the respective clinics. These exploratory retrospective analyses in the EMBER-MDD project will generate molecular “-omics” aggregated data via different laboratory and bioinformatic pipelines. We will analyze genomics, epigenomics, transcriptomics, proteomics and metabolomics based on biomaterials of the MDD patients, as well as the interactions between these “-omics”-modalities, e.g., methylation (meQTL) or expression (eQTL) quantitative trait loci: how genomics affects epigenomics and transcriptomics, etc. The aggregated data are not meant to allow any conclusions with direct clinical relevance, neither in terms of treatment efficacy nor in terms of tolerability for individual patients. Instead, the results of the EMBER-MDD study will be explorative, serve to generate new research hypotheses and will allow for an in-depth analysis focusing on the interplay of these multi-omic layers. Accordingly, our objectives focus on identifying biomarkers and signatures based on comprehensive omics analysis methods as detailed in the following sections. The expected results, their relevance, and the general meaning of the analyses will be explored and discussed in greater detail in the final section.

## Objectives

Our primary objectives are to identify individual omics and integrated multi-omics (hypothesis-free) biomarkers and signatures associated with risk of TR in MDD sufferers.

The secondary objectives are to identify individual omics and integrated multi-omics biomarkers for treatment response to ESK compared to TAU in MDD patients.

## Methods

Comprehensive omics analyses will be performed utilizing different biomaterials and laboratory pipelines followed by appropriate analysis pipelines as detailed below. Based on the retrospective and exploratory nature of the analyses we abstain from a priory power calculations and extensive corrections for multiple testing. Instead, to explore even putative (smaller) effects that might serve to generate new research hypotheses for future investigations, alpha-level will be set to 0.05 and FDR corrections performed only in selected omics analyses, e.g. transcriptomics (further details on statistics can be found in Online Resource “supplemental material”). Initially, each of the omics modalities will be analyzed individually using state-of-the-art bioinformatics analysis strategies, followed by joint analyses using novel bioinformatics multi-omics analysis strategies to integrate the different omics modalities as described below.

### Biomaterials

Biomaterials for the present study originate from MDD patients treated with either ESK (estimated *n* = 210) or TAU (estimated *n* = 210) and will be usable after these patients completed their treatments and left the clinic. Four different types of biomaterials will be available: whole blood, RNA-stabilized whole blood, plasma and serum. These types of biomaterials are based on venous blood samples taken from the patients at different time points (baseline and, if possible, during and after treatment), resulting in up to 5040 biosamples that are frozen and stored at − 80° C until usage in the present study (further details on biomaterials can be found in Online Resource “supplemental material”, section “Biomaterials”).

### Laboratory pipelines

**Fig. 1:**
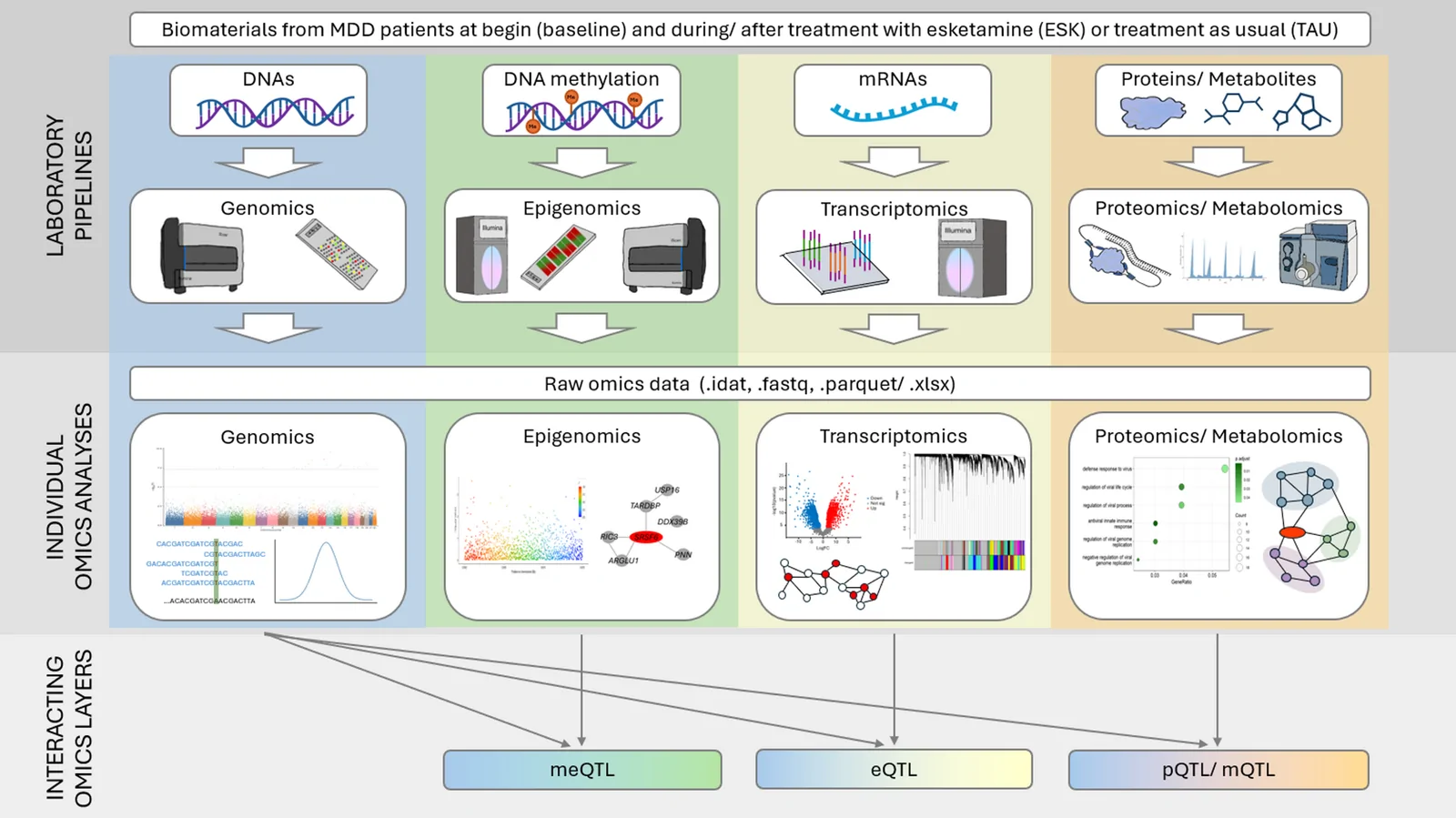
Illustration of EMBER-MDD study methods with laboratory pipelines, individual omics analyses and interacting omics layers. Biomaterials listed in the upper part are used for omic-specific platforms such as GSA v3.0+MD, MethylationEPIC v2.0 BeadChip, mRNA-NovaSeq, PEA-Olink^®^ and Biocrates MxP^®^ FIA-/ LC-MS/ MS. Resulting raw data undergo individual analyses for genomics (e.g. GWAS, PRS), epigenomics (e.g. EWAS), transcriptomics (e.g. DEG, WGCNA, PPI) and proteomics/ metabolomics (e.g. pathway and gene ontology analysis, PPI) as illustrated in the middle part of the figure. The lower part indicates downstream integrative analyses with PRS-based dimensionality reduction and QTL mapping (meQTL/ eQTL/ pQTL/ mQTL). (Illustration created with softwares GoodNotes 5 version 7.0.36 (system software OS Version 26.3.1 (a)) and PowerPoint version 2408 (system software Microsoft Office LTSC Professional Plus 2024)

Laboratory pipelines will utilize DNAs, DNA methylation, mRNAs, proteins and metabolites for genomics, epigenomics, transcriptomics and proteomics/ metabolomics platforms, respectively as illustrated in Fig. 1. Generated raw data will then run through individual omics analyses and interacting omics layers analysis (Fig. 1) as outlined below.

DNAs will be isolated from whole blood biosamples, undergo quality control (QC) measures and, if necessary, further purification and concentration steps to be used for genomics and epigenomics (DNA methylation) as described below and with details compiled in Online Resource “supplemental material” section “Laboratory pipelines”).

For genomics, only the baseline time point is used, that is, one DNA biosample per MDD patient (estimated *n* = 420). DNAs will be diluted and pipetted in randomized order based on patient’s treatment (ESK or TAU), sex and age on 96-well plates. Subsequent genome-wide genotyping will use Infinium™ GSA v3.0+MD arrays on HiScan array scanning systems (Illumina) resulting in raw genotyping data in “.idat” format (for details see Fig. 1 and Online Resource “supplemental material” section “Laboratory pipelines”).

For epigenomics, all available time points per MDD patient are used, that is, at least one DNA biosample at baseline and additional one or two DNA biosamples at time points within and/ or after treatment (estimated *n* = 420 up to *n* = 1260). DNAs will be diluted and pipetted in randomized order based on patient’s treatment (ESK or TAU), sex and age on 96-well plates with all “same-patient time points” positioned on the same plate. After bisulfite conversion, genome-wide DNA methylation screening will be performed using Infinium™ MethylationEPIC v2.0 BeadChip on HiScan array scanning systems (Illumina) resulting in raw data in “.idat” format (details: Online Resource “supplemental material” section “Laboratory pipelines”).

Total RNAs will be isolated from RNA-stabilized biosamples, undergo QC and RNA integrity measures, and different preparatory steps followed by specific library preparation to gain mRNAs libraries for subsequent mRNA-Seq as described below and detailed in Online Resource “supplemental material” section “Laboratory pipelines”.

For transcriptomics, all available time points per MDD patient will be used, that is, at least one mRNA library sample at baseline and additional one or two samples at time points within and/ or after treatment (estimated *n* = 420 up to *n* = 1260). Library samples are pipetted in randomized order based on patient’s treatment (ESK or TAU), sex and age on 96-well plates to be run e.g. on Illumina NovaSeq X or NovaSeq X Plus (both: Illumina) resulting in mRNA-Seq transcriptome profiling data in “.fastq” format (details: Online Resource “supplemental material” section “Laboratory pipelines”).

Plasma and serum biosamples include e.g. proteins, metabolites and lipids and provide the material to investigate proteomics and/ or metabolomics as detailed below.

For proteomics/ metabolomics, all available time points per MDD patient will be used, that is at least one biosample, preferably plasma, at baseline and one or two additional plasma biosamples collected during and/ or after treatment (estimated *n* = 420 up to *n* = 1260). Plasma aliquots will be processed using e.g. Olink^®^ and/ or Biocrates MxP^®^ technology, respectively, resulting in large-scale protein biomarker profiling data in “.parquet” format and/ or large-scale metabolite (lipids and small molecules) biomarker profiling data in “.xlsx” format (details: Online Resource “supplemental material” section “Laboratory pipelines”).

### Individual omics analyses

#### Genomic analyses

The analysis of genome-wide markers reflects the DNA in its purest form. The analysis of multiple genomic markers (e.g., from an Illumina GSA array, as described above) provides a basis from which to build upon. It provides a large number of single nucleotide variants (SNV’s), which can be used in a group-comparison in form of a genome-wide association study (GWAS) or, e.g., to estimate the genomic load of a number of traits in form of polygenic risk scores (PRS) as illustrated in Fig. 1. This analysis technique allows for a translational and multidimensional evaluation of genetic predisposition in the context of treatment resistance and treatment response. In addition, the basis provided by the SNV’s can also be used in interaction with other layers of the multi-omics spectrum, e.g., meQTL, eQTL, pQTL and mQTL (see below and Fig. 1). Finally, genome-wide markers provide a reliable estimate of statistically relevant confounding factors, such as ancestry.

#### Epigenomic analyses (DNA-methylation)

DNA-methylation is an epigenetic modification of the DNA that regulates its transcription. Epigenome-wide markers, such as the ones identified through the EPICv2 array can also be analyzed in a group-to-group comparison (i.e., epigenome-wide association analysis (EWAS) or focused on e.g., specific gene-regions, Fig. 1). In contrast to genomic markers, DNA-methylation can change over the course of time and in response to multiple exposures, such as treatment. Recent findings from our group suggest a response-related correlation between treatment with ketamine and DNA methylation [3]. However, similarly to the above-mentioned genomic markers, epigenome-wide DNA methylation can also be used to summarize underlying information, such as the reliable reconstruction of the cell-type composition of the venous blood sample (‘immunomethylomics’), which has also been linked to treatment response in previous research from our group [4]. As DNA methylation changes over time, but SNV’s do not, methylation quantitative trait loci (meQTL) provide an exquisite strategy to investigate whose DNA-methylation is most likely to change in the context of treatment, depending on their genetic predisposition. As the focus of the EMBER-MDD study is the methodological exploration of individual omics as well as multi-omic layers in the context of treatment, we see this method as particularly suiting for an in-depth understand of the relationship between genetic predisposition and DNA methylation as a dynamic biomarker.

#### Transcriptomic analyses

The analysis of genome-wide mRNA expression reflects the RNA transcripts produced by the genome at a specific point in time and provides an overview of gene activity through mRNA sequencing. This approach enables the identification of biomarkers that are differentially expressed under various conditions, allowing for the investigation of underlying molecular mechanisms and their changes over time. Transcriptome analyses comprise e.g. analysis of differentially expressed genes (DEGs) and/ or an investigation of biological networks based on pairwise correlations between variables, such as weighted gene coexpression network analysis (WGCNA) but also analyses of pathway enrichment and gene ontology as well as protein-protein interaction (PPI) (Fig. 1; details in Online Resource “supplemental material” section “Transcriptomic analyses”). As mRNA expression undergoes changes over time, but SNV’s do not, expression quantitative trait loci (eQTL) allow the investigation whose mRNA transcripts are most likely to change in the context of treatment, depending on their genetic predisposition.

#### Proteomic/ metabolomic analyses

The analysis of large-scale protein/ metabolite biomarker datasets, e.g. based on Olink^®^/ Biocrates MxP^®^ panels, reflects profiling more than 5,400 proteins/ 1,000 metabolites of top-level pathways such as inflammation, which is highly relevant in MDD neurobiology. These might reveal, for example, pathological mechanisms underlying MDD as well as might facilitate MDD subtype diagnosis via specific metabolite signatures (e.g. [5]). Proteomics/ metabolomics comprise analyses in a similar manner compared to transcriptomics as described above, that is e.g. differentially expressed protein/ metabolite analysis can be complemented by pathway enrichment and gene ontology analysis, WGCNA and PPI analysis as illustrated in Fig. 1 and detailed in Online Resource “supplemental material” section “Proteomic/ metabolomic analyses”. As the expression of proteins and metabolites changes over time, but again, SNV’s do not, protein (expression) quantitative trait loci (pQTL) and/ or metabolite (expression) quantitative trait loci (mQTL) allow the investigation whose proteins/ metabolites are most likely to change in the context of treatment, depending on their genetic predisposition.

### Integrative multi-omics analyses

**Fig. 2:**
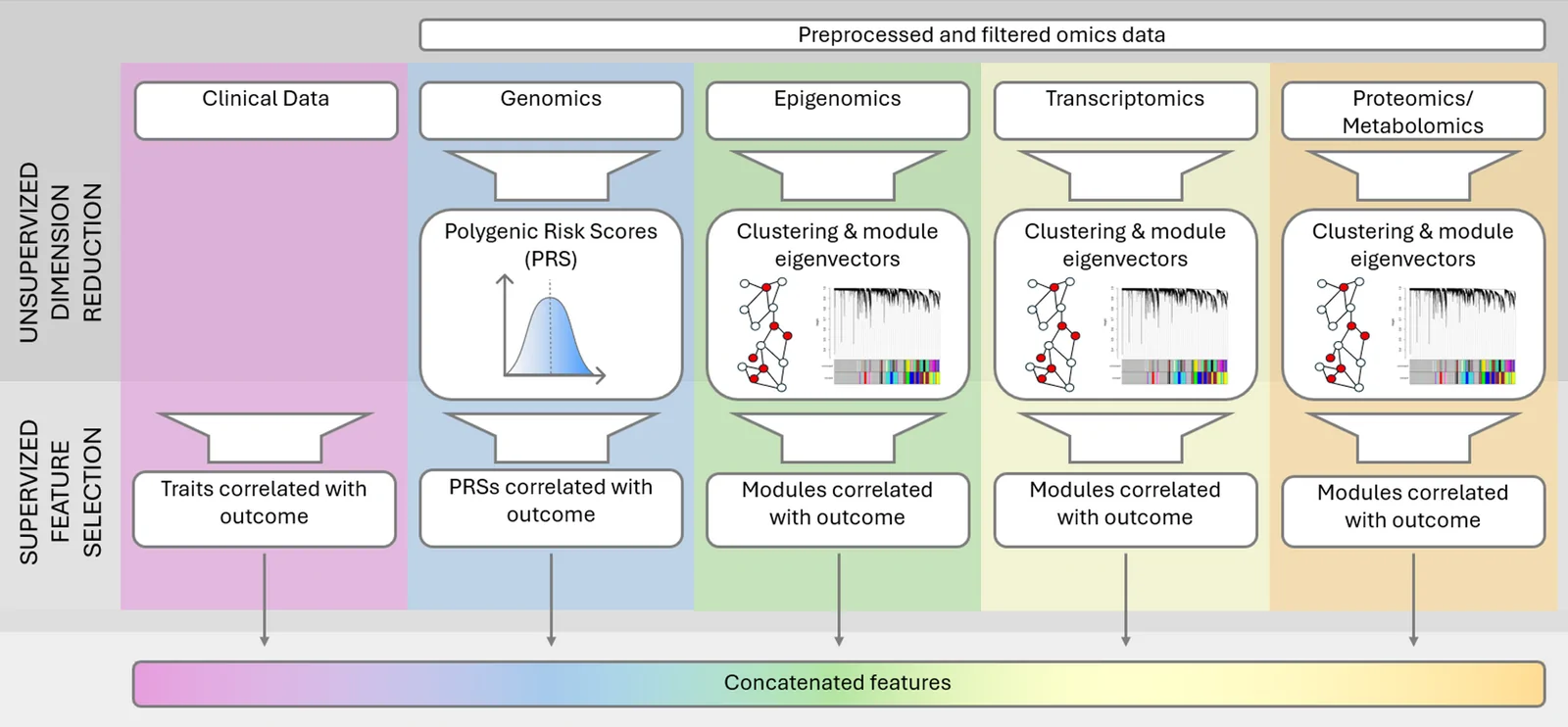
Illustration of multi-omics integration strategy. The integration is divided into two steps: (1) Unsupervised dimension reduction and (2) Supervised feature selection. In the first step, dimensionality of genomic data is reduced via polygenic risk scores; methylomic, transcriptomic and proteomic/ metabolomic data is clustered and each cluster represented by its first eigenvector. In the second step, for each data type, a subset of features is selected that correlate most with the outcome. Eventually features from each data type are standardized and concatenated. (Illustration created with softwares PowerPoint version 2408 (system software Microsoft Office LTSC Professional Plus 2024) and Figma (figma.com))

**Fig. 3:**
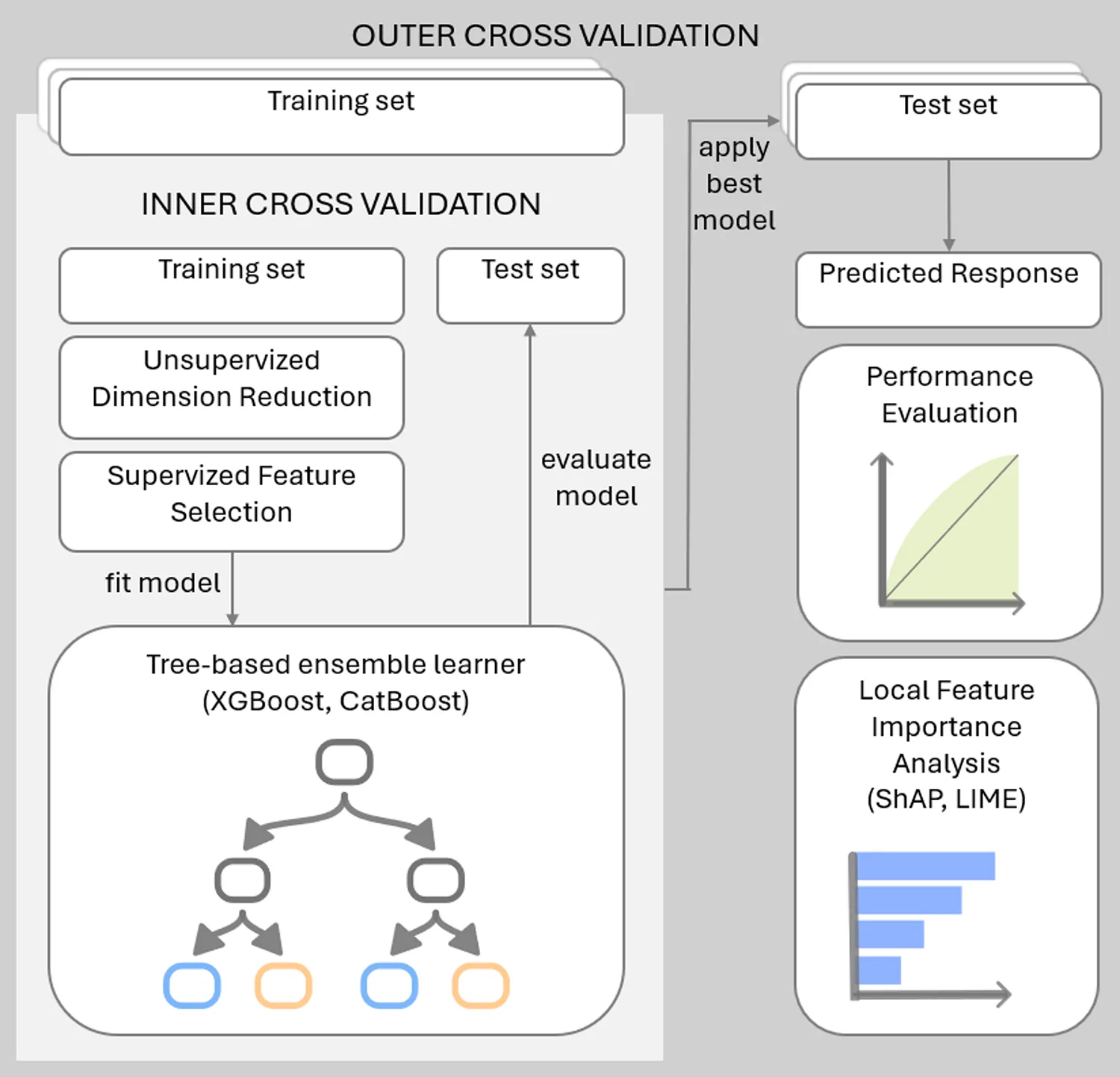
Illustration of ML workflow showing training and evaluation of the ensemble tree classifier in a nested cross-validation. Data integration (as shown in Fig. 2) and hyperparameter tuning is carried out in the inner cross-validation. The best performing model of the inner cross-validation is applied to the outer cross-validation’s test set to evaluate the model performance and analyze local feature importance. (Illustration created with softwares PowerPoint version 2408 (system software Microsoft Office LTSC Professional Plus 2024) and Figma (figma.com))

All available multi-omic layers will be integrated in a Machine Learning (ML) based analysis to gain a more holistic view on the biological processes and derive multi-omic signatures of treatment response. The overall pipeline will follow an intermediate fusion approach, first reducing the dimensionality of individual omic-layers, then concatenating them for downstream analysis (Fig. 2). Next, supervised classifiers will be trained to predict treatment outcome and TRD status after treatment (Fig. 3). None of the classifiers produced in the EMBER-MDD study are designed to be used in clinical decision making, but instead serve as a basis for applying methods of explainable AI to eventually generate hypotheses for potential multi-omic biomarkers. Machine Learning methods excel in finding complex non-linear patterns in integrated data, opposed to traditional statistics that are used for single omics and often assume distributions of input data, linearity of relationships and lack statistical power with increasing data complexity [6, 7, 8].

The available input variables comprise clinical (sociodemographic and symptomatic) data as well as genomic, methylomic, transcriptomic and proteomic/ metabolomic data from the previously described individual omic preprocessing pipelines. The high dimensionality of the omic layers compared to the number of instances is a common challenge, aggravated when handling multi-omic integration. This will lead to overfitting if unaddressed. Unsupervised dimension reduction will be achieved in a tailored mono-omic approach (Fig. 2), to account for the biological differences between data types and enable downstream interpretability of individual omic layers (e.g. pathway analyses) and evaluation of their predictive power. Dimensionality of genotypes will be reduced via polygenic risk scores (PRS) associated with treatment response including psychiatric, (immuno-)metabolic and cardiovascular phenotypes [9]. Methylomic, transcriptomic and proteomic/ metabolomic features will be clustered and each cluster represented by the eigenvector explaining most of the variance. For example, weighted correlation network analysis (WGCNA) clusters genes regarding their co-expression where each cluster can be represented by its respective eigengene. To retain balance of data modalities, further reduction of total input variable number will be achieved separately in each omic layer by selecting the features most correlated with the outcome. Eventually, features will be concatenated as input matrix for the prediction model.

An ensemble tree classifier will be trained in a nested stratified cross-validation to predict treatment response (Fig. 3). Ensemble tree algorithms are well suited as classifiers on integrated omics data as they are able to model complex non-linear patterns and have shown strong performance [10–12]. Due to inbuilt feature subsampling, tree-based algorithms can handle a comparatively high number of features, allowing for a wider search for biomarkers. The nested cross-validation will enhance generalizability by analyzing robustness of results on the outer folds and by separating hyperparameter tuning on the inner folds from estimating model performance (e.g. AUC, balanced accuracy). The hyperparameters contain model-specific parameters like tree depth, as well as parameters from omics integration such as thresholds related to number of clusters and total amount of features. It is important to note that the reduction and integration of features will be carried out on the train set and applied to the test set in each loop to avoid data leakage.

The primary goal of this integrative analysis is to apply methods of Explainable AI to the trained classifiers to uncover underlying biosignatures that contribute most to predictions and generate hypotheses for further biomarker studies. We will use local methods such as Shapley Additive Explanations (SHAP) [13] or Local Interpretable Model-agnostic Explanations (LIME) [14]. These approaches allow an understanding of patient-level feature importance and therefore facilitate subgroup analysis (such as esketamine vs. TAU). Due to the additive nature of ShAP, we could further evaluate the benefit of integrating multiple omics compared to training only on one modality. Long-term, this is relevant considering the trade-off between optimal model performance and feasibility and costs in a potential clinical context.

To further study the different mechanism behind esketamine and TAU, we will train meta algorithms (e.g. Conditional Average Treatment Effect (CATE)) to estimate treatment specific effects. Separate models on esketamine and TAU patients will be used to impute the unobserved treatment outcomes and calculate pseudo treatment effects for each patient. Based on these, treatment effect models will be trained and eventually combined to estimate the CATE. The goal is not to evaluate which treatment leads to better outcomes, but to understand mechanisms behind response of specific patients depending on their unique molecular profile, contributing to the development of tailored treatment to the individual person.

## Results—expected outcomes

This study will generate a deeply phenotyped, longitudinal, multi-omics resource in 420 adults with MDD sampled at baseline and follow-up during/ after treatment with esketamine (ESK) or treatment as usual (TAU). We expect three primary classes of deliverables: (i) validated prediction models; (ii) concise biosignatures derived from integrated omics; and (iii) systems-level maps that contextualise molecular signals with clinical outcomes. These results are enabled by harmonized genomics, DNA-methylation, transcriptomics, proteomics (and metabolomics) from whole blood, plasma and serum collected at predefined time points optimized for detecting early and standard-of-care response dynamics.

### Prediction and risk stratification

Using an intermediate-fusion pipeline, we will first reduce dimensionality within each omic layer (e.g., PRS for genotypes; WGCNA-derived eigengenes/ eigenproteins/ eigenmetabolites; feature selection by outcome correlation), then concatenate standardized features to train ensemble tree classifiers under nested, stratified cross-validation. We anticipate above-chance discrimination for (a) during (mid) treatment “early” improvement and (b) after (end of) treatment/ follow-up response, as well as probabilistic estimates of risk for treatment resistance (TR). Model explainability (SHAP/ LIME) will be used to quantify patient-level drivers of predictions and to compare the added value of multi-omics versus single-omic models. All models are for research use only and not for clinical decision-making.

### Biosignature generation

We expect to derive compact blood-based biosignatures—comprising a small set of stable markers or module scores—that reproduce across resampling folds and remain robust to batch/ covariate adjustment. Signatures will include both static (baseline) and dynamic (Δ from baseline to mid/ end of treatment) components to capture rapid and sustained biology. Explainable-AI rankings will guide down-selection to panels amenable to targeted assays in future validation work.

### Systems biology and network-level readouts

Pairwise and multi-layer network analyses (e.g., WGCNA modules, PPI overlays) will reveal coordinated changes across the transcriptome, proteome and metabolome that track symptom change. Integrative quantitative genetics (meQTL/ eQTL) will link inherited variants to methylation and expression states, clarifying how genotype shapes dynamic blood biology under treatment. Immunomethylomic deconvolution will provide cell-type context for differential signals, reducing confounding by shifts in leukocyte composition. Collectively, these analyses should highlight hub genes/ proteins and pathways for hypothesis-driven follow-up.

### Mechanistic insight into antidepressant action and resistance

By contrasting ESK with TAU across time, we anticipate delineating mechanisms consistent with rapid-acting antidepressant effects (e.g., synaptic/ plasticity and neuroimmune axes) alongside metabolic and stress-related programmes that differentiate responders from non-responders. Conditional average treatment effect (CATE) modelling will be used to estimate treatment-specific benefits at the individual level, enabling a mechanistic view of “who benefits from what” based on their unique molecular profile. These results will generate testable hypotheses on biological pathways predisposing to response or to resistance despite adequate treatment.

### Prediction of treatment response and resistance

For response, we expect early (mid treatment) transcriptomic/ proteomic/ metabolomic shifts to serve as leading indicators of later clinical improvement, informing minimal-panel predictors that remain robust after controlling for technical and clinical covariates. For resistance, we anticipate a baseline multi-omic signature—augmented by genotype-anchored regulatory links (meQTL/ eQTL)—that stratifies TR risk independently of treatment arm, with additional, treatment-specific resistance signatures emerging from CATE analyses.

### Use cases for multi-omics in this study

- Prediction: Cross-validated models combining PRS, methylomic modules, DEGs/ eigengenes, and protein/ metabolite features to estimate individual probabilities of early and standard-interval response.
- Biosignature generation: Explainable-AI-pruned marker sets (static and dynamic) suitable for future targeted assays.
- Systems biology: Multi-layer network maps (WGCNA→PPI) to prioritise hubs/ pathways for mechanistic follow-up.
- Understanding mechanisms: meQTL/ eQTL to trace genotype→epigenome→transcriptome→protein/ metabolite cascades that differ by treatment arm and outcome.
- Predicting treatment response: Early Δ-omics features that anticipate end of treatment outcome and refine patient-level expected benefit under ESK vs. TAU via CATE.
- Predicting treatment resistance: Baseline multi-omic risk profiles that identify patients likely to remain symptomatic under conventional strategies, informing intensified approaches to test in future studies.

### Planned outputs

We will report (i) model performance under nested CV with modality-ablation analyses; (ii) ranked, minimally redundant biosignatures with stability metrics; and (iii) integrative pathway/ network summaries linking molecular modules to clinical trajectories. All findings will be interpreted within the constraints of a non-interventional biomarker study and positioned to guide future prospective validation.

## Discussion

In this protocol, we outline a non-interventional, multi-omics biomarker study embedded in the Psych-STRATA programme, designed to identify molecular signatures of treatment resistance (TR) and treatment response in adults with MDD treated with esketamine (ESK) or treatment as usual (TAU). This work addresses an urgent clinical need: a substantial proportion of patients develop treatment-resistant depression (TRD) with enduring functional impairment despite adequate pharmacotherapy [1]. While the Psych-STRATA consortium as a whole aims to develop a stratified treatment algorithm across severe mental illnesses [2], the present EMBER-MDD study specifically focuses on leveraging longitudinal, blood-based multi-omics and advanced machine learning (ML) to generate hypotheses on biological mechanisms underlying TR and response in MDD.

### Positioning within current research

To date, most biomarker research in MDD and TRD has focused on single modalities (e.g., candidate genes, peripheral inflammatory markers, or single-layer transcriptomics) or cross-sectional designs, often in modest samples and without robust validation strategies. These approaches have yielded putative markers and pathways (e.g., immune and metabolic dysregulation, synaptic plasticity), but have not translated into clinically actionable tools [1]. The EMBER-MDD study extends existing work in several ways. First, it capitalises on harmonised sampling and treatment paradigms within INTENSIFY and its mirror study OBS-TR, enabling a relatively large, deeply phenotyped cohort (*n* ≈ 420) with pre-defined time points aligned with early and standard-of-care treatment response. Second, it combines genomic, epigenomic (DNA methylation), transcriptomic, proteomic and metabolomic data, allowing us to study both static predisposition and dynamic molecular change. Third, integrative analyses via PRS, meQTL/ eQTL, and multi-layer network approaches build a quantitative bridge from inherited variation to treatment-related shifts in gene expression, immune profiles and circulating proteins/ metabolites, extending previous immunomethylomics work in MDD (van Assche et al. [4]). Finally, the ML and explainable AI (XAI) framework is explicitly designed not only to classify response versus non-response, but also to highlight candidate biosignatures and mechanistic patterns that can be followed up in subsequent studies.

### Conceptual and methodological strengths

A central strength of this protocol is the longitudinal, multi-omics design spanning baseline, mid-treatment and end-of-treatment/ follow-up time points. This enables us to investigate both baseline predictors of TR risk and early “Δ-omics” indicators of impending response or non-response. In contrast to purely cross-sectional omics studies, this design directly targets temporal dynamics, which may be particularly relevant for rapid-acting antidepressants such as ESK and for early stratification during treatment. Recent work from our group suggests that ketamine-related changes in DNA methylation may be detectable in blood, underlining the value of longitudinal epigenomic profiling in this context [3].

Second, the study uses high-density, genome-/ omic-wide platforms (GSA, EPIC v2, mRNA-Seq, Olink^®^, Biocrates MxP^®^) to comprehensively capture genetic, epigenetic, transcriptomic, proteomic and metabolomic variation. Genotypes provide a stable anchor for downstream integrative analyses, facilitating PRS-based dimensionality reduction and QTL mapping (meQTL/ eQTL/ pQTL/ mQTL). The use of immunomethylomic deconvolution further allows us to reconstruct blood cell-type composition from DNA methylation data, mitigating confounding by leukocyte shifts and linking immunological context to treatment response [3, 4].

Third, the integrative ML pipeline has been designed to reflect both statistical rigor and biological interpretability. An intermediate fusion approach—with tailored, modality-specific dimensionality reduction followed by feature selection and subsequent concatenation—helps to manage the high p/n ratio typical of multi-omics datasets. Nested stratified cross-validation reduces the risk of optimistic bias by separating feature selection and hyperparameter tuning from performance estimation. The choice of ensemble tree-based methods is well suited to potentially non-linear interactions between omics and clinical variables, while XAI methods (e.g. SHAP, LIME) allow us to map model predictions back to interpretable features, both at the group and the patient level. This is crucial in a field where black-box models, without mechanistic insight, are unlikely to have enduring impact.

Fourth, the study sits within the broader Psych-STRATA framework [2], which provides an overarching infrastructure of harmonized assessments, shared analytic pipelines and cross-disorder comparisons (SCZ, BD, MDD). This facilitates downstream replication and extension of findings, including the possibility to test whether MDD-specific biosignatures are distinct from, or partially shared with, signatures of TR in other severe mental illnesses.

### Clinical and translational implications

Although our models are explicitly not intended for immediate clinical decision-making, the study is designed to generate several clinically relevant hypotheses. First, baseline multi-omics profiles may help to stratify patients by their probability of achieving adequate response under conventional treatment strategies, thereby informing the design of future trials that test intensified or alternative treatments in high-risk groups. Second, early (mid-treatment) shifts in epigenomic, transcriptomic, or proteomic/ metabolomic signatures may function as leading indicators of later clinical improvement, potentially enabling shorter, more efficient clinical trials and future clinical algorithms that adapt treatment earlier in non-responders. The relevance of circulating metabolites as potential biomarkers has already been demonstrated in plasma-based studies distinguishing MDD cases from controls (Liu et al. [5]), supporting the rationale for including metabolomics.

Third, by comparing ESK and TAU at the molecular level, the study aims to delineate treatment-specific mechanisms, rather than merely assessing which treatment performs better. CATE modelling will allow us to explore heterogeneity of treatment effects as a function of individual molecular profiles, providing a mechanistic lens on “who benefits from what.” In the longer term, such insights could support precision prescribing strategies, where biological profiles help prioritise rapid-acting agents versus conventional options in selected patients.

Finally, the generation of compact biosignatures—concise sets of markers or eigengene/ module scores robust across resampling—could inform development of future targeted assays (e.g., multiplex panels) that are more feasible in routine care than full omics profiling. This would be an important step towards translating complex multi-omics findings into practical tools.

### Limitations and challenges

Several limitations of the present protocol warrant consideration. First, despite the depth of molecular phenotyping, the study remains a non-interventional biomarker project based on blood-derived samples. As such, causal inferences about mechanisms of antidepressant action or resistance will be limited. Peripheral markers may only partially reflect central nervous system processes, and interpretation of brain-relevance will rely heavily on pathway and network analyses, QTL links, and convergent evidence from the literature [1].

Second, although the expected sample size (*n* ≈ 420) is substantial for a deeply phenotyped, multi-omics dataset in MDD, it is modest relative to the dimensionality of genome-wide and –omics data. Even with strong regularization, nested cross-validation and careful avoidance of data leakage, there remains a non-trivial risk of overfitting and unstable feature selection. Therefore, predictive performance estimates must be interpreted cautiously, and multi-omics signatures should be considered preliminary until replicated in independent cohorts or within other arms of Psych-STRATA [2].

Third, not all patients are expected to contribute complete data across all omics modalities and time points. Missingness due to sample quality, patient burden, or logistical constraints may introduce selection bias in integrative analyses if, for example, more severely ill or less adherent patients are underrepresented in particular omics layers. Planned strategies such as imputation, sensitivity analyses and robust feature selection can mitigate but not fully eliminate such biases.

Fourth, generalizability may be constrained by the inclusion criteria and treatment context. The sample consists of adult MDD patients treated within specific clinical services and protocols (ESK vs. second-line antidepressants), which may differ from other health care systems, age groups, or treatment strategies (e.g., psychotherapy-focused pathways, neuromodulation). The external validity of discovered biosignatures will therefore need examination in diverse clinical settings.

Fifth, while the combination of INTENSIFY and OBS-TR broadens ecological validity, it may introduce heterogeneity in treatment trajectories, concomitant medications, and support structures. Such factors, along with site and batch effects, will need careful modelling and adjustment. Residual confounding by lifestyle factors (smoking, diet, activity), comorbidities, and psychosocial stress is also likely and may obscure or mimic biological signatures of TR.

Finally, the predictive and stratification framework raises important ethical questions. Even though models in this study are for research only, future translation of TR risk estimates into practice would require careful evaluation of patient acceptability, potential stigma, informed consent, and safeguards around data privacy and fairness (e.g., avoiding systematic disadvantages to particular demographic groups). These aspects fall outside the scope of the current protocol but should be considered from the outset when planning long-term implementation.

### Future directions

The current study is primarily discovery-oriented and hypothesis-generating. A key next step will be the external validation of identified biosignatures and prediction models in independent cohorts, ideally with harmonized phenotyping and sampling schedules. Cross-disorder analyses within Psych-STRATA could test whether specific modules (e.g., immune, metabolic, synaptic-plasticity related) are shared across TR in MDD, SCZ and BD, or whether distinct patterns emerge by diagnosis [2]. Moreover, integration of multi-omics with other modalities—such as neuroimaging, digital phenotyping or microbiome data—could further refine mechanistic hypotheses and predictive performance.

From a translational standpoint, downselection to pragmatic biomarker panels will be crucial. Candidate signatures derived from XAI-informed rankings can be evaluated in targeted follow-up studies using lower-cost assays and simplified sampling schemes. In parallel, experimental work in cellular or animal models may help to test causality for high-priority genes, pathways or networks identified in this study.

## Conclusion

In summary, the EMBER-MDD study within Psych-STRATA is designed to generate an integrated, longitudinal, blood-based multi-omics resource in adults with MDD undergoing ESK or TAU, and to apply state-of-the-art analytic methods to identify candidate biomarkers and biosignatures of treatment response and resistance. By combining genomic predisposition with dynamic epigenomic, transcriptomic, proteomic and metabolomic changes, and embedding these in an explainable ML framework, the study aims to bridge the gap between complex molecular data and clinically meaningful hypotheses. While constraints related to design, sample size, tissue specificity and generalisability must be acknowledged, the planned analyses are expected to provide an important foundation for future validation studies and, ultimately, for the development of stratified, mechanism-informed treatment strategies in MDD.

## Supplementary Information

Below is the link to the electronic supplementary material.

Supplementary Material 1

## Data Availability

No datasets were generated or analysed during the current study.
