# Supplementary material for: Esketamine multi-omic biomarker evaluation in major depressive disorder (EMBER-MDD): concept, objectives and methodologies of a non-clinical investigator-initiated study
Source: Eur Arch Psychiatry Clin Neurosci. 2026 Jun 30;276(7):3469–79. doi: 10.1007/s00406-026-02306-x (PMC13562347; doi:10.1007/s00406-026-02306-x)
Supplement: Supplementary file 1 [file 406_2026_2306_MOESM1_ESM.docx]

Supplemental material to:

**Esketamine multi‑omic biomarker evaluation in major depressive disorder (EMBER‑MDD): concept, objectives and methodologies of a non‑clinical investigator‑initiated study.**

*European Archives of Psychiatry and Clinical Neuroscience.*

C. Hohoff^1^, T. Lange^1^, L. Steinbach^1^, S. M. Wissing^1^, E. van Assche^1^, B. T. Baune^1,2,3^

*^1^Department of Psychiatry, University of Muenster, Muenster, Germany*

*^2^Department of Psychiatry, Melbourne Medical School, University of Melbourne, Melbourne, Australia*

*^3^ Department of Psychiatry, The Florey Institute of Neuroscience and Mental Health, Melbourne, Australia*

Corresponding author: B. T. Baune, bernhard.baune@uni-muenster.de

**Methods**

***Biomaterials***

Whole blood biosamples contain K3 EDTA (e.g., Greiner Bio-One, Frickenhausen, Germany; Sarstedt, Nümbrecht, Germany) anticoagulated and stabilized venous blood that provide the material to isolate DNA by standard procedures as mentioned below.

RNA-stabilized whole blood biosamples contain different chemicals such as RNA*later*™ solution (Thermo Fisher Scientific), PAXgene^®^ Blood RNA Tubes additive (Qiagen) or S-Monovette^®^ RNA Exact preparation solution (Sarstedt). These different chemicals stabilize RNAs by preventing RNA degradation and by minimising unwanted “post-blood-collection” induction of gene expression. Depending on the chemicals, appropriate standard methods and kits will be used for RNA isolation as detailed below.

Plasma and serum biosamples contain the liquid fraction of whole blood after centrifugal separation of solid (e.g. cellular) components of the venous blood collected in e.g. K3 EDTA tubes or Serum tubes or SerumGel tubes (Greiner Bio-One; Sarstedt). These liquid fractions are available in aliquots (appropriate portions) per patient and time point. Plasma and serum aliquots include e.g. proteins, metabolites and lipids and provide the material to investigate proteomics as detailed below.

***Laboratory pipelines***

DNAs will be isolated from whole blood biosamples, e.g. using QIAamp DNA Blood Mini- or Midi-Kits (Qiagen, Hilden, Germany) as recommended by the manufacturer. Isolated DNAs will undergo quality control (QC) measures via microvolume spectrophotometric or fluorescence-based methods (Qubit; Thermo Fisher Scientific/Life Technologies, Darmstadt, Germany). If necessary, further purification and concentration will follow utilizing Amicon Centrifugal Filter Devices (Amicon® Ultra 0.5ml 3K; Merck/Millipore, Darmstadt, Germany). DNAs are of optimum quality with purity ratios of 1.8-2.0 for A_260_/A_280_, 1.7-2.2 for A_260_/A_230_ and concentrations > 60ng/µl for subsequent usage in genomics and epigenomics as detailed below.

For genomics, DNAs will be diluted to 60 ng/µl in aliquots containing 15 µl corresponding to final amounts of 900 ng DNA per patient and are pipetted on 96-well plates for subsequent genome-wide genotyping using Infinium^TM^ Global Screening Array-24+ v3.0 (GSA v3.0+MD; Illumina, San Diego, CA, USA) on HiScan array scanning systems (Illumina) following manufacturer’s instructions. Resulting genotyping data in “.idat” format will then be further processed to perform genome-wide association studies (GWAS) as detailed in the main text.

For epigenomics, DNAs will be diluted to a range of 60 to 70 ng/µl in aliquots containing 15 to 18 µl corresponding to final amounts of 900 to 1260 ng DNA per patient and pipetted on 96-well plates. After bisulfite conversion, genome-wide methylation screening will be performed using Infinium^TM^ MethylationEPIC v2.0 (930k) BeadChip (Illumina) on HiScan array scanning systems (Illumina) following manufacturer’s instructions. Resulting epigenome profiling data in “.idat” format will undergo QC assessment and further processing to perform epigenome-wide association studies (EWAS) as detailed in the main text.

Total RNAs will be isolated depending on the chemicals used for RNA-stabilisation. For example, for RNA*later*™ stabilised biosamples, the RiboPure™-Blood Kit (Thermo Fisher Scientific) is recommended and will be used followed by RNA cleaning and concentrating using the TURBO DNA-free™ Kit (Thermo Fisher Scientific) and Amicon® Ultra 0.5 mL 3K Centrifugal Filter Devices (Merck) following manufacturer’s instructions. PAXgene^®^ stabilised biosamples will be processed with the PAXgene^®^Blood miRNA Kit for the purification of miRNA and total RNA as recommended by the manufacturer (Qiagen). S-Monovette^®^ RNA Exact stabilised biosamples will be utilised for RNA isolation using the NucleoSpin^®^ RNA Blood Midi kit (Macherey-Nagel, Düren, Germany) following manufacturer’s instructions. Isolated and purified RNAs will undergo QC and RNA integrity measures, e.g. on NanoDrop 8000 (Thermo Fisher Scientific) and 2100 expert bioanalyzer with Eukaryote Total RNA Nano chip (Agilent, Santa Clara, CA, USA) or TapeStation 4200 with RNA ScreenTape or high sensitivity RNA ScreenTape (Agilent). RNA integrity numbers (RIN) > 6 indicate limited degradation and sufficient quality, RIN > 7 indicate good quality and RIN > 8 optimum quality 4. Concentrations and amounts of RNAs will be chosen dependent on the downstream chemicals and kits for mRNA library preparation, e.g., 4 ng/µl in 50 µl aliquots corresponding to final input amounts of 200 ng RNA per patient and time point. RNAs will undergo different preparatory steps, for example mRNA enrichment and globin depletion will be followed by library preparation using e.g. the Watchmaker Genomics System (e.g., mRNA Capture Kit, RNA LibPrep Kit, Polaris Depletion Kit rRNA/Globin, IDT xGen Stubby Adapter UDI; all from Watchmaker Genomics DACH, Germany) and the instructions of the manufacturer resulting in mRNA libraries.

For transcriptomics, final mRNA libraries can be run for example on Illumina NovaSeq X or NovaSeq X Plus (both: Illumina) with e.g. Illumina NovaSeq™ X Series 1.5B Reagent Kit (200 Cyc, Illumina) as recommended by the manufacturer. Resulting mRNA-Seq transcriptome profiling data in “.fastq” format will undergo QC assessment and further processing to perform transcriptome-wide association studies (TWAS) as detailed in the main text and below.

Plasma and serum biosamples include e.g. proteins, metabolites and lipids and provide the material to investigate proteomics and metabolomics.

For proteomics, plasma aliquots will be processed using e.g. Olink^®^ Explore HT panels (Olink^®^ Proteomics AB, Uppsala, Sweden; Olink^®^: Part of Thermo Fisher Scientific) according to the manufacturer’s protocols, combining proximity extension assay (PEA) technology with next generation DNA sequencing (NGS) readouts on e.g. Illumina NovaSeq X Plus (Illumina) platform. The resulting large-scale protein biomarker profiling dataset in “.parquet” format will undergo QC assessment and further processing to perform high-throughput proteomics and proteome-wide association studies (PWAS) as detailed in the main text and below.

For metabolomics, plasma aliquots will be processed using e.g. Biocrates MxP®500 Kit XL (biocrates life sciences ag, Innsbruck, Austria) based on flow injection analysis tandem mass spectrometry (FIA-MS/MS) and liquid chromatography tandem mass spectrometry (LC-MS/MS) on a SCIEX 5500 QTrap mass spectrometer and/ or an Agilent 1290 Infinity II liquid chromatography system coupled with SCIEX 5500 QTrap. Resulting large-scale metabolite (lipids and small molecules) biomarker profiling data in “.xlsX” format will undergo QC assessment and further processing to perform high-throughput metabolomics as detailed in the main text and below.

***Transcriptomic analyses***

Our mRNA-Seq data analysis pipeline starts with extensive QC to check raw data (.fastq) quality and to clean up sequencing reads. This includes the use of trimming tools like Trim Galore [1], Cutadapt [2] or Trimmomatic [3] to remove technical sequences (e.g., adapters), bases with low quality, contaminating sequences and sequencing errors. Trimming thus creates a solid foundation for subsequent alignment of reads to a reference genome, for example using STAR [4] or Bowtie/Bowtie2 [5]. Next, the number of reads mapped to each transcript will be counted to quantify relative expression by determining the relative amounts of individual transcripts in the samples utilising e.g., HTSeq-count [6], FeatureCounts [7] or RSEM [8]. This results in a count table (counts per gene for each sample) that will be further processed by exploratory preliminary analysis, e.g. to obtain an initial overview or to identify potential patterns or outliers. Exploratory preliminary analysis includes various techniques for clustering, reducing data dimensions, identifying potential covariates and batch factors and visualising the data. For example, hierarchical cluster analysis (HCA) examines the similarity between samples based on their expression profiles, which helps identifying potential data subgroups and visualising variability between samples. HCA uses a distance metric (e.g. Euclidean distance, Pearson correlation) and a linking method (e.g. Ward, Average) by clustering tools (e.g. pheatmap [9]; ComplexHeatmaps [10]). Principal component analysis (PCA) reduces the dimensionality of the data and visualise the variability between samples. This can help to identify potential outliers and understand the sample variability using scaling tools such as PCAtools [11] or FactoMineR [12]. Potential phenotypic covariates (e.g., age, biological sex, education years, disease severity) are extracted from the available clinical data and are analysed via different parametric or non-parametric statistical tests (e.g. Pearson/Spearman correlation, T-test/Mann-Whitney U test). Potential technical covariates (e.g. batch effects due to different sequencing kits, plates, runs, platforms) and hidden/unknown effects are extracted from the available sequencing data and are analysed as well via different parametric or non-parametric statistical tests (see above). If needed, batch effects can be corrected for using multiple algorithms such as SVAseq [13], RUVseq [14] or ComBatseq [15]. In subsequent DEG analysis, gene expression differences between experimental conditions will be examined based on a created design matrix that took into account the experimental conditions and relevant covariates and/or batch effects. The read counts will be normalised using methods such as DESeq2's normalization or trimmed mean of M-values (TMM) to account for technical biases associated with sequencing batches, gene length, library size, and other protocol-specific artifacts [16]. DEGs are identified utilising commonly used tools like DESeq2 [17], edgeR [18] or Limma-voom [19], employing the corresponding model type (e.g. negative binomial model; empirical Bayes). To minimise the risk of false positive results, the resulting DEGs can then be corrected for multiple testing using methods such as false discovery rate (FDR). DEGs can be further investigated with respect to potentially underlying or involved biological mechanisms that might be influenced by the experimental conditions. Therefore, the list of DEGs will be used to identify enriched biological pathways by means of path enrichment tools such as gProfiler [20] or clusterProfiler [21]. These tools test for over-representation of particular pathways in a gene list compared to the common representation than would be expected by chance using databases like Gene Ontology [22], KEGG [23] or Reactome [24], including as well a statistical correction of multiple testing. Complex relationships between genes can be investigated with WGCNA [25] which identifies highly correlated (co-expressed) pattern in count data and clusters genes into modules with similar expression patterns and weighted adjacency matrices. So-called module eigengenes or intramodular hub genes can be calculated for correlation networks between modules and with clinical traits. The functional significance of identified co-expressed module or hub genes can be further investigated with PPI analysis using WGCNA-modules or other relevant gene-lists as input. PPI results will be retrieved from established databases such as STRING [26] or BioGRID [27] and a PPI network will be constructed and visualised e.g. by Cytoscape [28] to allow analysis of interaction patterns.

***Proteomic/ metabolomic analyses***

Proteomic/ metabolomic analyses comprise normalization, QC assessment and further processing, e.g. using the OlinkAnalyze R package [29] or the Perseus software [30], respectively, in a similar manner compared to transcriptome analyses as described above. That is, steps of normalization, QC, exploratory preliminary analysis, potential covariate/batch effect analyses and analysis of differentially expressed proteins/ metabolites can be complemented by pathway enrichment and gene ontology analysis, WGCNA and PPI analysis as detailed below.

Protein raw data counts will be converted into Normalized Protein Expression (NPX) values based on internal and external controls, Log2 transformation and normalization across plates. Extensive QC analyses further evaluate e.g. controls and sample quality relative to controls per individual plate as well as assay quality, intra- and inter-plate percent coefficient of variance and putative signal deviation from median values of controls. QCs can further be visually checked using e.g. PCA of controls and samples or different QC plot functions implemented in OlinkAnalyze, for example NPX distribution per sample per panel or potential outlier identification by IQR vs. sample median per panel. Subsequently, HCA and PCA analyses, analogue to those used in the transcriptomics pipeline (details above), examine similarities or variabilities between samples, respectively. Further, potential phenotypic covariates (e.g., age, biological sex) and technical covariates (e.g. batch effects) are analysed via different parametric or non-parametric statistical tests (see above) and can be corrected for if necessary (e.g. via SVAseq, RUVseq, ComBatseq; see above). Subsequent DEP analysis investigates protein expression differences between study conditions based on experimental design using e.g., Welch Two Sample T-test with FDR-adjustment for multiple testing followed by visualization e.g. by volcano plot (OlinkAnalyze). DEPs can be further examined utilizing e.g. gProfiler or clusterProfiler, both with databases like Gene Ontology, KEGG or Reactome, to identify potentially involved biological mechanisms, followed by e.g. WGCNA and PPI, with databases like STRING or BioGRID as well as visualisation e.g. by Cytoscape, to identify complex relationships and interaction patterns between genes (all analogue to transcriptomic analyses, see above).

Metabolite raw data concentrations will be analysed using e.g. Perseus software using processing and analysis steps analogue to the above-mentioned steps for protein data analyses.

**References:**

1. Krueger F, James F, Ewels P, Afyounian E, Weinstein M, Schuster-Boeckler B, Hulselmans G, sclamons (2023). Zenodo. FelixKrueger/TrimGalore: v0.6.10 - add default decompression path (0.6.10). <https://doi.org/10.5281/zenodo.7598955>
2. Martin M (2011) Cutadapt removes adapter sequences from high-throughput sequencing reads. EMBnet Journal.  [https://doi.org/10.14806/ej.17.1.200](%20https://doi.org/10.14806/ej.17.1.200)
3. Bolger AM, Lohse M, Usadel B (2014) Trimmomatic: a flexible trimmer for Illumina sequence data. Bioinformatics 30:2114-2120.  [https://doi.org/10.1093/bioinformatics/btu170](%20https://doi.org/10.1093/bioinformatics/btu170)
4. Dobin A, Davis CA, Schlesinger F, Drenkow J, Zaleski C, Jha S, Batut P, Chaisson M, Gingeras TR (2013) STAR: ultrafast universal RNA-seq aligner. Bioinformatics 29:15-21. [https://doi.org/10.1093/bioinformatics/bts635](%20https://doi.org/10.1093/bioinformatics/bts635)
5. Langmead B, Trapnell C, Pop M, Salzberg SL (2009) Ultrafast and memory-efficient alignment of short DNA sequences to the human genome. Genome Biol. [https://doi.org/10.1186/gb-2009-10-3-r25](%20https://doi.org/10.1186/gb-2009-10-3-r25)
6. Anders S, Pyl PT, Huber W (2015) HTSeq: a Python framework to work with high-throughput sequencing data. Bioinformatics 31:166-169. <https://doi.org/10.1093/bioinformatics/btu638>
7. Liao Y, Smyth GK, Shi W (2014) featureCounts: an efficient general purpose program for assigning sequence reads to genomic features. Bioinformatics 30:923-930. [https://doi.org/10.1093/bioinformatics/btt656](%20https://doi.org/10.1093/bioinformatics/btt656)
8. Li B, Dewey CN (2011) RSEM: accurate transcript quantification from RNA-Seq data with or without a reference genome. BMC Bioinformatics 12:323. [https://doi.org/10.1186/1471-2105-12-323](%20https://doi.org/10.1186/1471-2105-12-323)
9. Kolde R (2025) pheatmap: pretty heatmaps. R package version 1.0.13.
10. Gu Z, Eils R, Schlesner M (2016) Complex heatmaps reveal patterns and correlations in multidimensional genomic data. Bioinformatics 32:2847-2849. [https://doi.org/10.1093/bioinformatics/btw313](%20https://doi.org/10.1093/bioinformatics/btw313)
11. Blighe K, Brown AL, Carey V, Hooiveld G, Lun A (2023) PCAtools: Everything Principal Components Analysis. R package version 2.20.0. <https://doi.org/10.18129/B9.bioc.PCAtools>
12. Lê S, Josse, J, Husson F (2008). FactoMineR: An R Package for Multivariate Analysis. Journal of Statistical Software 25: 1–18. <https://doi.org/10.18637/jss.v025.i01>
13. Leek JT (2014) svaseq: removing batch effects and other unwanted noise from sequencing data. Nucleic Acids Res 42:161. <https://doi.org/10.1093/nar/gku864>
14. Risso D, Ngai J, Speed TP, Dudoit S (2014) Normalization of RNA-seq data using factor analysis of control genes or samples. Nat Biotechnol 32:896-902. <https://doi.org/10.1038/nbt.2931>
15. Zhang Y, Parmigiani G, Johnson WE (2020) ComBat-seq: batch effect adjustment for RNA-seq count data. NAR Genom Bioinform. <https://doi.org/10.1093/nargab/lqaa078>
16. Stupnikov A, McInerney CE, Savage KI, McIntosh SA, Emmert-Streib F, Kennedy R, Salto-Tellez M, Prise KM, McArt DG (2021) Robustness of differential gene expression analysis of RNA-seq. Comput Struct Biotechnol J. 19:3470-3481. <https://doi.org/10.1016/j.csbj.2021.05.040>
17. Love MI, Huber W, Anders S (2014) Moderated estimation of fold change and dispersion for RNA-seq data with DESeq2. Genome Biol 15:550. <https://doi.org/10.1186/s13059-014-0550-8>
18. Robinson MD, McCarthy DJ, Smyth GK (2010) edgeR: a Bioconductor package for differential expression analysis of digital gene expression data. Bioinformatics. 26:139-140. <https://doi.org/10.1093/bioinformatics/btp616>
19. Law CW, Chen Y, Shi W, Smyth GK (2014) voom: precision weights unlock linear model analysis tools for RNA-seq read counts. Genome Biol. [https://doi.org/10.1186/gb-2014-15-2-r29](%20https://doi.org/10.1186/gb-2014-15-2-r29)
20. Reimand J, Isserlin R, Voisin V, Kucera M, Tannus-Lopes C, Rostamianfar A, et al (2019) Pathway enrichment analysis and visualization of omics data using g:Profiler, GSEA, Cytoscape and EnrichmentMap. Nat Protoc 14:482-517 [https://doi.org/10.1038/s41596-018-0103-9](%20https://doi.org/10.1038/s41596-018-0103-9)
21. Yu G, Wang L, Han Y, He QY (2012) clusterProfiler: an R package for comparing biological themes among gene clusters. OMICS 16:284-287. [https://doi.org/10.1089/omi.2011.0118](%20https://doi.org/10.1089/omi.2011.0118)
22. Ashburner M, Ball CA, Blake JA, Botstein D, Butler H, Cherry JM, et al (2000) Gene Ontology: tool for the unification of biology. Nat Genet 25:25-29. [https://doi.org/10.1038/75556](%20https://doi.org/10.1038/75556)
23. Kanehisa M, Furumichi M, Tanabe M, Sato Y, Morishima K (2017) KEGG: new perspectives on genomes, pathways, diseases and drugs. Nucleic Acids Res. <http://doi.org/10.1093/nar/gkw1092>
24. Fabregat A, Sidiropoulos K, Viteri G, Forner O, Marin-Garcia P, Arnau V, et al (2017) Reactome pathway analysis: a high-performance in-memory approach. BMC Bioinformatics 18:142. <https://doi.org/10.1186/s12859-017-1559-2>
25. Langfelder P, Horvath S (2008) WGCNA: an R package for weighted correlation network analysis. BMC Bioinformatics. <https://doi.org/10.1186/1471-2105-9-559>
26. Szklarczyk D, Kirsch R, Koutrouli M, Nastou K, Mehryary F, Hachilif R, et al (2023) The STRING database in 2023: protein-protein association networks and functional enrichment analyses for any sequenced genome of interest. Nucleic Acids Research. <https://doi.org/10.1093/nar/gkac1000>
27. Stark C, Breitkreutz BJ, Reguly T, Boucher L, Breitkreutz A, Tyers M (2006) BioGRID: a general repository for interaction datasets. Nucleic Acids Res, <https://doi.org/10.1093/nar/gkj109>
28. Shannon P, Markiel A, Ozier O, Baliga NS, Wang JT, Ramage D, Amin N, Schwikowski B, Ideker T (2003) Cytoscape: a software environment for integrated models of biomolecular interaction networks. Genome Research 13: 2498-2504. [https://doi.org/10.1101/gr.1239303](%20https://doi.org/10.1101/gr.1239303)
29. Nevola K, Sandin M, Guess J, Forsberg S, Cambronero C, Pucholt P, Zhang B, Sheikhi M, Diamanti K, Kar A, Conze L, Chin K, Topouza D, Pollo S, Dong K, Hodén K, Eriksson P, Moloney N, Lötstedt B, Sprecher E, Barbagallo J, Mansson O, Caster O, Olink (2025). OlinkAnalyze: Facilitate Analysis of Proteomic Data from Olink. R package version 4.3.2. <https://doi.org/10.32614/CRAN.package.OlinkAnalyze>
30. Tyanova S, Temu T, Sinitcyn P, Carlson A, Hein MY, Geiger T, Mann M, Cox J (2016). The Perseus computational platform for comprehensive analysis of (prote)omics data. Nat Methods 13:731-740. <https://doi.org/10.1038/nmeth.3901>
